# Location-Dependent Empirical Thresholds for Quantitative Trait Mapping

**DOI:** 10.1534/g3.112.003517

**Published:** 2012-09-01

**Authors:** Jason LaCombe, Benjamin McClosky, Steven Tanksley

**Affiliations:** Nature Source Genetics, Ithaca, New York 14850

**Keywords:** QTL, empirical threshold, adjusted *P*-value, local error rate, multiple hypothesis testing

## Abstract

The Churchill-Doerge approach toward constructing empirical thresholds has received widespread use in the genetic mapping literature through the past 16 years. The method is valued for both its simplicity and its ability to preserve the genome-wide error rate at a prespecified level. However, the Churchill-Doerge method is not designed to maintain the local (comparison-wise) error rate at a constant level except in situations that are unlikely to occur in practice. In this article, we introduce the objective of preserving the local error rate at a constant level in the context of mapping quantitative trait loci in linkage populations. We derive a method that preserves the local error rate at a constant level, provide an application via simulation on a *Hordeum vulgare* population, and demonstrate evidence of the relationship between recombination and location bias. Furthermore, we indicate that this method is equivalent to the Churchill-Doerge method when several assumptions are satisfied.

The accurate detection of significant quantitative trait loci (QTL) remains an area of active research in the genetics mapping community (Falke and Frisch 2011; Manichaikul *et al.* 2007; Müller *et al.* 2011; Wei *et al.* 2010). In studying linkage populations, the process of performing a genome scan to detect QTL may be interpreted within a hypothesis-testing framework. Traditionally, at each point in the genome a statistical test is performed, resulting in a *P*-value representing the evidence for the presence of a QTL at that location. As the number of analysis points grows, so does the number of statistical tests performed, and the chance of incorrectly declaring a QTL at a given marker (committing a Type-I error) increases.

The multiple-testing problem is well studied in both the scientific and statistical literature (Dudoit and van der Laan 2007; Falconer and Mackay 1996; Lehmann and Romano 2005; Lynch and Walsh 1998; Seaman and Muller-Myhsok 2005; Storey and Tibshirani 2003). While several different types of error rates may be considered (*e.g.*, the false positive rate or false discovery rate), this article is concerned with methods that provide control of the genome-wide error rate (GWER).^2^ Of such methods, by far the most widely used is the permutation-based method of Churchill and Doerge (1994).

We first briefly review the steps involve in the application of the Churchill-Doerge (CD) method (Churchill and Doerge 1994). For demonstration purposes, we take the example of single marker analysis, although the discussion is also applicable to interval mapping. For each marker, a user seeks to compare the observed log-of-odds (LOD) score with some underlying distribution in order to calculate a *P*-value representing the level of statistical evidence present for a genetic effect. Churchill and Doerge (1994) propose comparing the LOD score at a marker to the empirical distribution of the maximum LOD score across the genome, under the null hypothesis of no QTL present in the genome (or a QTL present but not linked with the given marker.) The empirical distribution of the max LOD score under the null is estimated by repeatedly sampling the magnitude of the max LOD score across random permutations of the phenotype data.

The CD method is attractive for several reasons. First, the method is simple to apply and is currently implemented in a range of popular QTL analysis software packages: R/qtl (Broman *et al.* 2003), GenStat (VSN International 2011), MapQTL (Van Ooijen 2009), QTL Cartographer (Basten *et al.* 2002), etc. Second, the method is empirically driven, and so will more accurately represent the characteristics of the observed data than other methods relying on parametric assumptions. Finally, the method can be demonstrated to strongly control the GWER at a prespecified level.

However, under certain conditions, the CD method may result in upwardly or downwardly biased estimates for the Type-I error in specific regions of the genome—a property we refer to as location bias. Such behavior may be expected to occur when the location-conditioned distributions of the LOD (not max LOD) score are not identical. Several relevant examples are presented in Peirce *et al.* (2007, 2008), and the respective authors provide evidence for the consideration of linkage as a possible causal factor of location bias.

The primary focus of this article is in deriving a multiple-testing procedure that not only preserves the GWER but also maintains the local error rate at a constant level across the genome. The method presented possesses the property that Type-I errors are no more likely to occur at a given locus than at an alternative. For simplicity, we confine our attention to the technique of marker regression, although this work is easily extended to any approach in which a finite number of single hypotheses are considered (*e.g.*, interval mapping on a finite grid).

The derived method is motivated by the following considerations. Suppose that the distribution of the maximum LOD score is not identical across the genome. That is, for two non-identical markers, it is possible that the distribution of the maximum LOD score at the first marker is not identical to the distribution of the max LOD score at the second marker. For a given marker, we propose a comparison of the associated LOD score with the distribution of the maximum LOD score at that marker. Thus the comparison is with a conditional—rather than a marginal—distribution.

Proceeding with the comparison of the observed LOD scores with the appropriate conditional max LOD distributions, we seek to construct a threshold that preserves the GWER at a prespecified level, while maintaining the local error rate at a constant level.

## NOTATION AND METHODS

In this section, we introduce the mathematical notation relevant to the remainder of this work, and we present a derivation of the proposed method.

Denote the number of markers in the genome scan as $n_{m}$, and define $n_{obs}$ to be the number of individuals for which we possess phenotype information. Throughout the remainder of this article, we assume that the observed genotype data are complete (*i.e.*, no missing values); a discussion on the implications of missing data are presented in File S1. We represent an observation of the maximum LOD as *M*, where *M* is defined as the pair (δ,λ), with *δ* the magnitude of the max LOD value and *λ* the location (marker index) of the max LOD value. A threshold *T* is defined as a sequence of non-negative values indexed from 1 to $n_{m}$.

We next present the candidate method—referred to as the location-dependent threshold (LDT) method—for constructing empirical thresholds similar to those of Churchill and Doerge (1994), while accounting for local properties of the genome. Our goal is to construct a threshold $T_{LDT}\;$such that for a desired GWER *γ*, $P\left\{\delta >T_{LDT},\;\;\lambda =i\right\}=\frac{\gamma }{n_{m}}.$ The LDT method relies on generating observations of *M* under random permutations of the observed phenotypes. However, unlike the CD method, which relies on the marginal distribution of *δ*, the proposed procedure necessitates the derivation of estimates of the empirical distribution function of *δ* conditioned on *λ*. Estimating these conditional distributions requires that we generate many more samples (the number of samples denoted as $n_{p}$) than the Churchill-Doerge procedure, enough so that $n_{p}P\left\{\lambda =i\right\}\approx 1000$ for all *i*. Although the computational burden is significantly increased, the additional resources are needed to accurately estimate and account for location bias.

To begin, let *T* be an arbitrary threshold and fix *γ* at the desired GWER. For each *i* define $\alpha^{i}={\left[n_{m}P\left\{\lambda =i\right\}\right]}^{-1}$, and a function $H_{i}$ such that $H_{i}\left(T^{i}\right)$ is the unique value satisfying

$$P\left\{\delta^{i}>T^{i}|\lambda =i\right\}=\frac{P\left\{\delta^{i}>H_{i}\left(T^{i}\right)|\lambda =i\right\}}{\alpha^{i}}.$$

Note that, for a collection of $n_{m}$ markers, $H_{i}\left(T^{i}\right)$ exists if and only if $\gamma <\underset{i}{\min}\left\{\frac{1}{\alpha^{i}}\right\},$ otherwise, the equality in the above equation does not hold. Let $T_{\lambda }$ be a sequence of location-dependent threshold values such that $P\left\{\delta^{i}>T_{\lambda }^{i}|\lambda =i\right\}=$ γ. Then $H(T_{\lambda })$, the sequence generated by the evaluation of $H_{i}\left(T_{\lambda }^{i}\right)$ for all *i*, is a threshold that preserves the GWER at level *γ* (see *Appendix*).

The role of the function *H* is to adjust the threshold in the presence of location bias. For example, when $\alpha^{i}$ is greater than one, there exists a positive bias away from location *i*; by dividing by $\alpha^{i}$ we are shrinking the numerator in Equation 1 toward zero, and so must decrease the threshold accordingly. Thus, we adjust for the presence of a positive location bias by decreasing the corresponding significance threshold. Similarly, we adjust for the presence of a negative location bias by increasing the threshold.

### Example of controlling the local error rate

**Figure 1:**
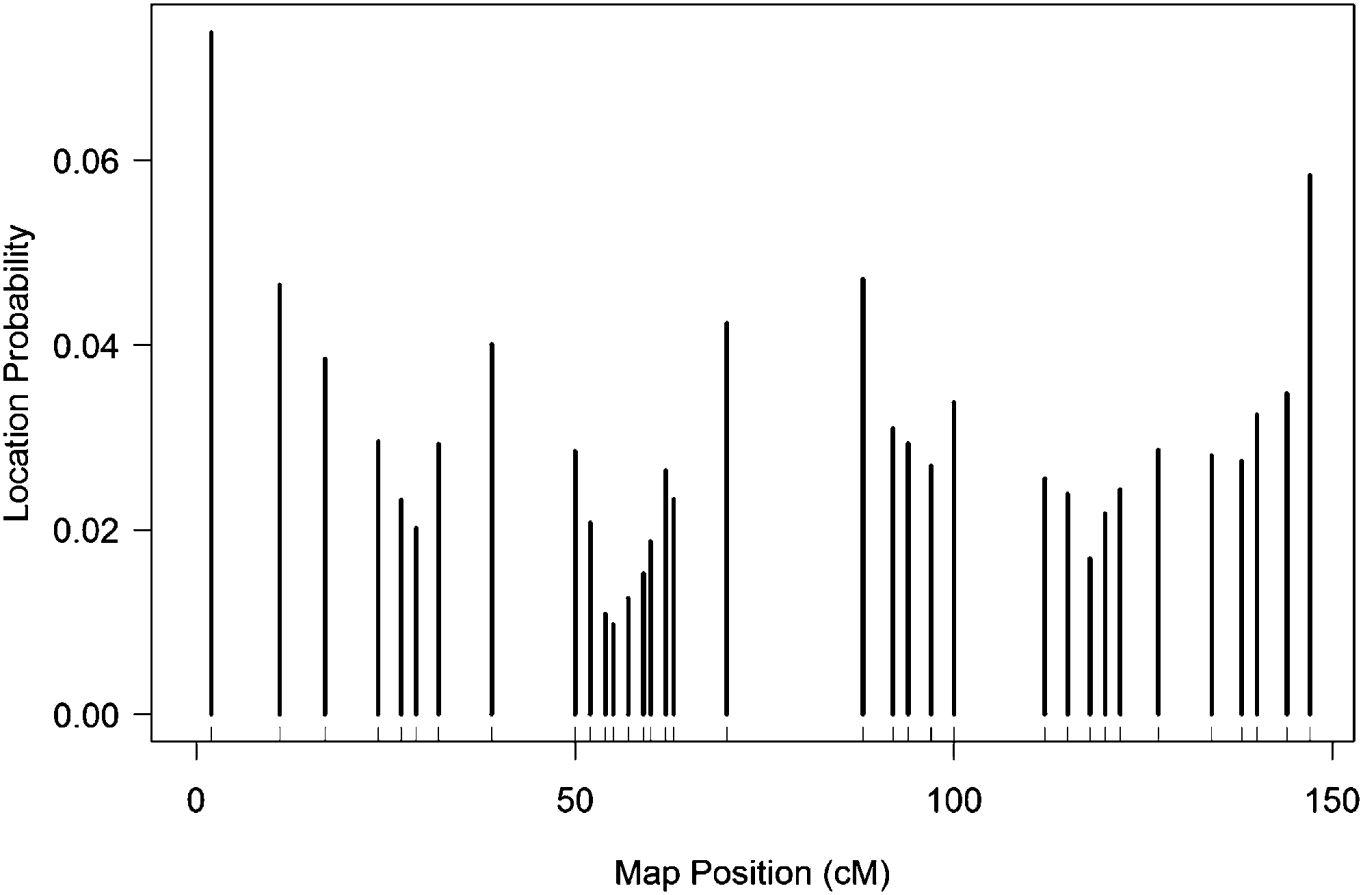
Plot of the empirical location distribution of *λ* for a single chromosome of a synthetic double-haploid population of 200 individuals, with 34 markers. Observe that the distribution of *λ* has a tendency toward smaller probabilities in areas of dense markers, while *λ* has larger probabilities in areas of sparse markers.

As an example, suppose we observe genotype information at 34 markers for 200 individuals from a backcross population. A demonstration of the presence of location bias in this synthetic population is given in Figure 1.

**Figure 2:**
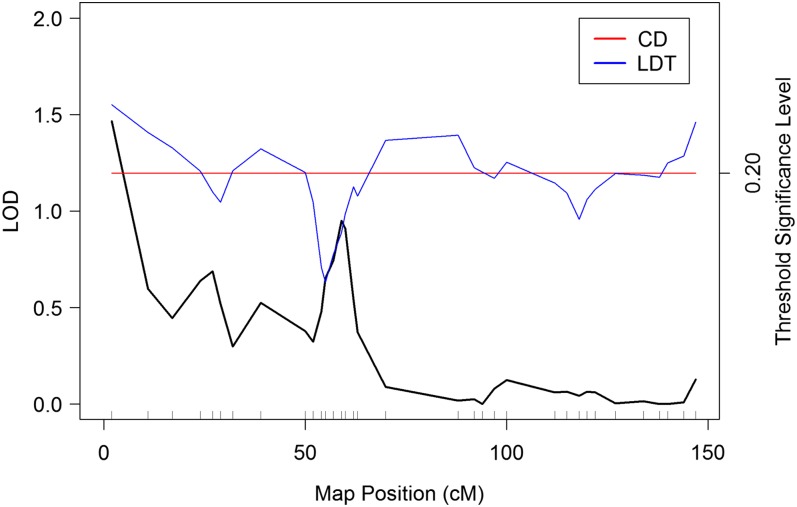
Plot of the LOD score constructed for the synthetic population associated with Figure 1. Thresholds preserving the GWER at 20% are displayed for both the CD method and the LDT method. Note that the CD threshold remains constant across the genome, while the LDT threshold varies with location.

Consider the problem of constructing a 20% genome-wide threshold for the sample of maximum LOD scores corresponding to this population; such a construction is given in Figure 2. The figure illustrates the difference between the CD method (the construction of a constant genome-wide threshold that places 20% of the observed max LOD scores above the line) and the LDT method, which distributes 20% of the observed max LOD scores above the line, while assigning a constant percentage above the threshold at each marker, and thus varies with location.

Although the CD method controls the GWER in a marginal sense, *e.g.*, $\text{P}\left\{\delta >T_{CD}\right\}=\gamma$, it is not difficult to show that the CD method does not control the joint location-magnitude in a local sense (in the presence of location bias.) For the proposed method, the GWER is preserved both in a global and local sense with regard to the joint location-magnitude:

$$\text{P}\left\{\delta >T_{LDT}\;,\;\;\lambda =i\right\}=\frac{\gamma }{n_{m}}.$$

### Physical linkage

In accordance with Peirce *et al.* (2008), one potential justification for the presence of location bias is the existence of physical linkage between markers, *i.e.*, differences in recombination rates between markers. We illustrate the plausibility of such a hypothesis by considering the following experiment.

**Figure 3:**
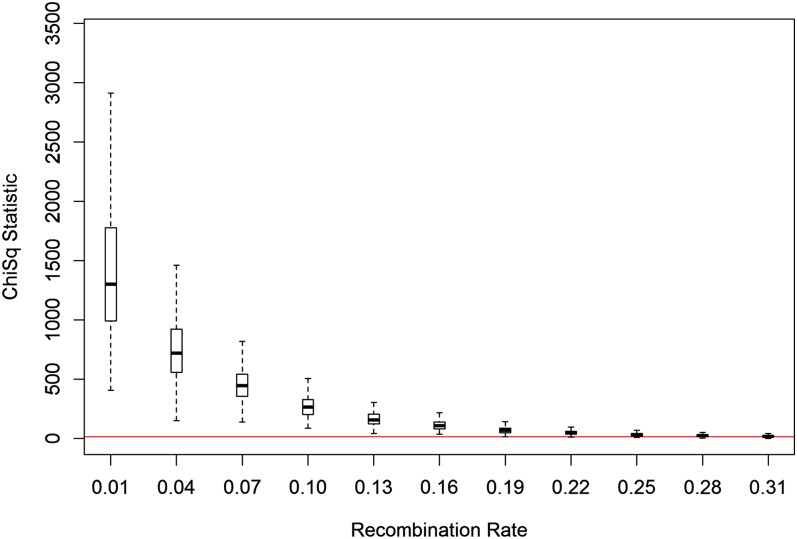
Plot of the relationship between recombination rate and the presence of location bias. The figure illustrates that as the recombination rate between markers increases, the deviance from uniformity decreases, indicating a decrease in location bias. The red line indicates the 95^th^ percentile for a Chi-squared statistic with nine degrees of freedom.

Suppose that we have a set of 10 equally spaced markers, representing observations for 200 individuals from a backcross population. Consider a sequence of 11 simulations, such that for each simulation the recombination rate between each marker is incremented by 0.03 units (starting at a recombination rate of 0.01.) Thus for the first simulation, we observe 10 markers, with recombination rates of 0.01, while in the final simulation, the rates are increased to 0.31. For each simulation, we compute vectors of the alpha-values for 300 independent samples of genotype data. For each individual sample, we perform a Chi-squared test for deviation from the uniform distribution and present the associated box-plots in Figure 3. The results support the hypothesis that as the recombination rate increases, the less severe the deviation of the alphas vectors from uniformity (the presence of location bias decreases.) As the LDT method accounts for location bias, we can then make the case that the method at least partially accounts for differences in recombination rates between markers.

### Barley example

In this section, we illustrate the performance of the proposed method applied to a double haploid empirical barley population derived from a cross of ‘Steptoe’ and ‘Morex’ (Kleinhofs *et al.* 1993). Phenotype and genotype data were downloaded from the GrainGenes website (Mather 1995). For this example, we considered the average of grain yield across 16 environments (P. M. Hayes *et al.* 1993) for 150 double haploid lines, with genotype data observed for 223 markers set across seven chromosomes.

**Figure 4:**
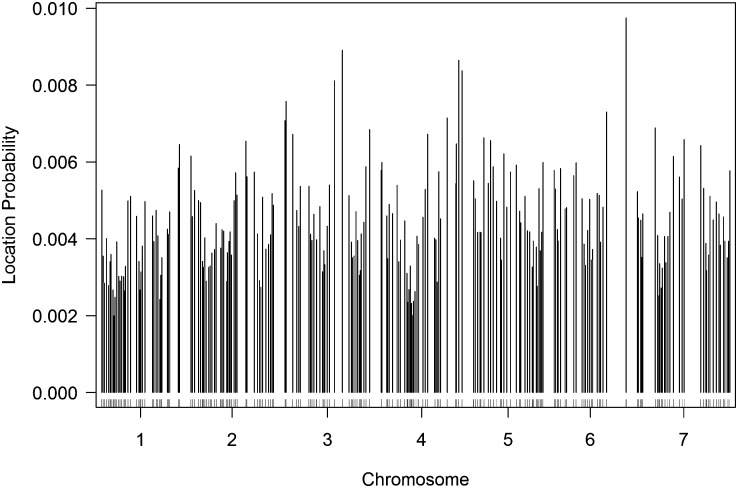
Plot of the empirical location distribution of *λ* for the average yield trait of the barley population.

For the comparison, a single sample consisting of 1,000,000 observations of *M* was generated and used to construct both the CD and LDT thresholds. Figure 4 indicates the presence of an observable location bias. We calculate thresholds at the 0.01, 0.10, and 0.40 levels for both the CD and LDT methods. The data (File S3) and R code (File S2) for the analysis are provided.

## DISCUSSION

**Figure 5:**
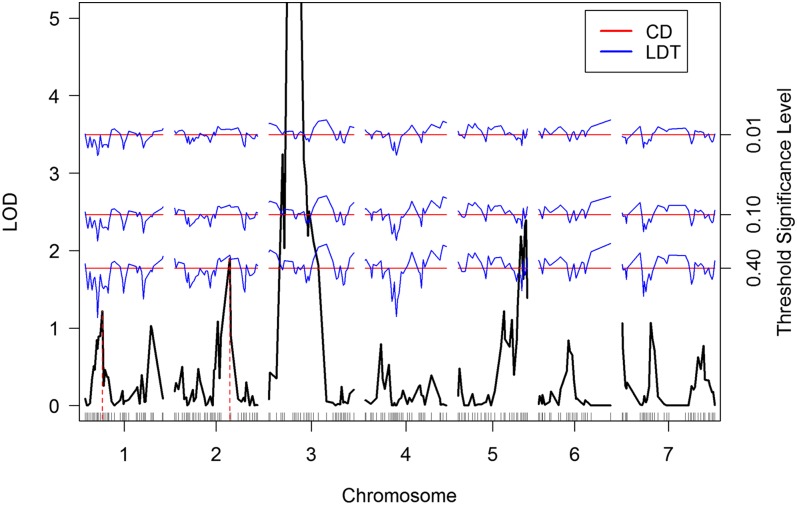
Plot of the LOD score constructed for the average yield trait of the barley population. Significance thresholds at the 0.01, 0.10, and 0.40 levels are displayed for both the CD method (in red) and the LDT method (in blue). The red, dashed, vertical lines indicate the markers associated with the highest LOD peaks on chromosomes 1 and 2, respectively. The range of the plot is restricted to better illustrate the performance of the two methods.

The results of the threshold constructions for the barley example are presented in Figure 5. We first observe that the LDT thresholds appear centered about the thresholds provided by the CD method. This behavior is consistent with Equation 1, to the extent that if the location-conditioned distributions are all identical, the average of the LDT threshold across the genome will be the CD threshold.

Although the CD threshold may be viewed as the approximate average of the location-dependent threshold, in the event that an investigator desires to construct adjusted *P*-values, *e.g.*, (Lystig 2003), the two methods produce different results. However, it is important to note that the LDT thresholds only preserve the GWER at levels in the range $\min_{i}\left\{\frac{1}{\alpha^{i}}\right\}$ to 1.

For example, consider the marker associated with the highest LOD peak on chromosome 2 (Figure 5). The CD-adjusted *P*-value is 0.3176, while the LDT-adjusted *P*-value is larger, at 0.4352. Similarly, marker on chromosome 1 has a CD-adjusted *P*-value of 0.8370, while the LDT-adjusted *P*-value is smaller at 0.5676 (Figure 5). In comparing the relative evidence for a QTL linked to the marker on chromosome 1 *vs.* chromosome 2 (*i.e.*, the ratio of the adjusted *P*-values), the LDT method results in twice as much relative evidence as that derived by the use of the CD method.

While the presented location-dependent methodology has the potential to provide more accurate QTL detection (in the sense of preserving the local Type-I error rate at a constant level), the methodology is not without criticism. First, it should be recognized that the number of required permutations ($n_{p})\;$is much greater than the number required for the CD method. In accordance with Churchill and Doerge (1994), we suggest enough so that there are at least 1000 observations of the max LOD score at each marker location. Thus, while Churchill and Doerge suggest a sample size of 1000 for their method (to maintain precision at the 0.05 level), the LDT method requires a suggested number of $5000\cdot n_{m}\;$samples to account for moderate location bias.

Also, one might suggest that for markers that never or rarely attain a maximum LOD score, it is not reasonable to compare observed LOD score with the distribution of the maximum. Rather, an investigator might choose to compare the observed score to a mixture distribution of LOD score order statistics, *e.g.*, Simonsen and McIntyre (2004).

Furthermore, as previously indicated, the LDT thresholds only preserve the GWER at levels within a range determined by the observed location bias $\left(0,\min_{i}\left\{\frac{1}{\alpha^{i}}\right\}\right)$. When controlling the GWER at levels that approach the maximum of this range, the magnitude of the LDT-adjusted *P*-values will be driven entirely by the location parameter, as opposed to a mixture of the location and magnitude parameters.

Although the above criticisms are valid, it remains desirable to consider QTL detection methods—within the hypothesis-testing framework—that preserve both the GWER as well as the local error rate at a constant level. Failing to account for location bias leaves an investigator susceptible to a varying and unquantified level of bias in the estimation of the Type-I error rate for different regions of the genome. It should also be noted that, in the event that the location-conditioned distributions are identical and no location bias exists, the LDT threshold is equivalent to the CD threshold. More generally, the proposed location-adjusted *P*-values are obtained through simple linear transformations of the *P*-values for the location-conditioned distributions (see *Appendix*.)

## CONCLUSION

The method of Churchill and Doerge (1994) provides investigators with an approach to QTL detection that maintains control of the GWER at a prespecified level. However, the presence of location bias has implications for the accurate assessment of local Type-I error rates throughout the genome.

The introduction of the LDT method provides investigators with an approach that controls the GWER, while also maintaining the local error rate at a constant level. Through simulation, we have demonstrated evidence that the method accounts for the presence of physical linkage. We have provided an application of the method to an empirical barley data set to better illustrate the method’s performance on data derived under realistic experimental conditions. Finally, we have observed that, in the presence of identical location-conditioned distributions and the absence of location bias, the method is equivalent to the approach of Churchill and Doerge.

## Supplementary Material

Supporting Information

## APPENDIX

In this appendix, we prove that the LDT method preserves the GWER at a prespecified level *γ*. First, choose *γ* such that $\gamma \;<\min_{i}\left\{\frac{1}{\alpha^{i}}\right\}.$Then,

$$\begin{array}{l} P\left\{\delta >H\left(T_{\lambda }\right)\right\}=\sum_{i=1}^{n_{m}}P\left\{\delta^{i}>H_{i}\left(T_{\lambda }^{i}\right)\;|\lambda =i\right\}P\left\{\lambda =i\right\}\\ =\sum_{i=1}^{n_{m}}\frac{P\left\{\delta^{i}>H_{i}\left(T_{\lambda }^{i}\right)\;|\lambda =i\right\}}{\alpha^{i}}\alpha^{i}P\left\{\lambda =i\right\}\\ =\sum_{i=1}^{n_{m}}\frac{P\left\{\delta^{i}>H_{i}\left(T_{\lambda }^{i}\right)\;|\lambda =i\right\}}{\alpha^{i}}\frac{1}{n_{m}}=\frac{1}{n_{m}}\sum_{i=1}^{n_{m}}P\left\{\delta^{i}>T_{\lambda }^{i}\;|\lambda =i\right\}=\frac{1}{n_{m}}\sum_{i=1}^{n_{m}}\gamma \\ =\gamma . \end{array}$$

In addition, the adjusted *P*-values for the method can be represented as a point-wise linear transform of the *P*-values for the location-conditioned distributions. Let $L^{i}$ represent the LOD score observed at marker *i*. Then,

$$\begin{array}{l} \;p_{i}=\;P\left\{\delta^{i}>H_{i}^{-1}\left(L^{i}\right)\;|\lambda =i\right\}=\frac{P\left\{\delta^{i}>H_{i}\left(H_{i}^{-1}\left(L^{i}\right)\right)\;|\lambda =i\right\}}{\alpha^{i}}\\ =\frac{P\left\{\delta^{i}>L^{i}\;|\lambda =i\right\}}{\alpha^{i}}. \end{array}$$
