# Supplementary material for: Location-Dependent Empirical Thresholds for Quantitative Trait Mapping
Source: G3 (Bethesda). 2012 Sep 1;2(9):1035–9. doi: 10.1534/g3.112.003517 (PMC3429917; doi:10.1534/g3.112.003517)
Supplement: Supporting Information [file supp_2_9_1035__index.html]

Supporting Information 

# Location-Dependent Empirical Thresholds for Quantitative Trait Mapping

## Supporting Information for LaCombe, McClosky, and Tanksley, 2012

**Files in this Data Supplement:**

- Supporting Information - Files S1-S3 and Figures S1 and S2 (PDF, 228 KB)
- File S1 - Extended Methods including Figures S1 and S2 (PDF, 209 KB)
- File S2 - R code for Barley Analysis (.zip, 2 KB)
- File S3 - Barley Data for Barley Example (.csv, 71 KB)
