# Supplementary material for: Location-Dependent Empirical Thresholds for Quantitative Trait Mapping
Source: G3 (Bethesda). 2012 Sep 1;2(9):1035–9. doi: 10.1534/g3.112.003517 (PMC3429917; doi:10.1534/g3.112.003517)
Supplement: Supporting Information [file supp_2.9.1035_003517SI.pdf]

File S1

Extended Methods

In this section we illustrate—via two simulations—the impact of missing data on the presence of location bias. Specifically we will generate the data according to two different modeling assumptions: Missing Completely At Random (MCAR) and Not Missing At Random (NMAR). The MCAR assumption entails that the events that cause missing data are independent of the observed and unobserved variables of interest. The NMAR assumption entails that a dependency exists between the events that cause missing data and either one or both of the observed or unobserved variables.

In both simulations we a consider set of 41 equally spaced markers (spaced at 5cM), representing observations for 200 individuals from a backcross population. For each missing data model we perform a series of 50 marginal simulations, such that the range of missing data varies from one to fifty percent. For each simulation we compute vectors of the alpha-values for 1000 independent samples of genotype data with the associated percentage of missing data. For each individual sample, we perform a Chi-squared test for deviation from the uniform distribution.

In generating data under the MCAR assumption, missing data is distributed uniformly across the matrix of observed genotypes. As there are an infinite number of ways to generate data under the NMAR assumption, we choose an approach that seems reasonable for our purposes. In generating NMAR data, we sample marker indices from a discretized version (to the nearest marker index) of the following mixture of normal distributions:

$$\frac{1}{3}\mathrm{N}(13.5{,}6) + \frac{2}{3}\mathrm{N}(27.5{,}6), \qquad (2)$$

and sample (without replacement) the row index of the associated marker in which to insert the missing data.

The results of the MCAR and NMAR analyses are presented in Figure S1 and Figure S2, respectively. In each figure, the points plotted represent the average of the Chi-squared statistic over the observed 1000 independent samples for the associated percentage of missing data. In both cases the level of missing data did not significantly increase or decrease the presence of location bias (MCAR p-value: 0.3341, NMAR p-value: 0.3369; from the regression of missing data percentage on the Chi-squared statistic for the associated missing data model), though location bias was present regardless.

One potential reason for the absence of a missing data effect on location bias is that the Max LOD location and magnitude were sampled using Haley-Knott regression at the markers (which reduces to standard marker regression in the complete absence of missing data.) The choice to use Haley-Knott regression is driven by the method's ability to correct for missing data (and so would be used in the presence of such), as well as its implementation in standard QTL analysis software, such as R/qtl.

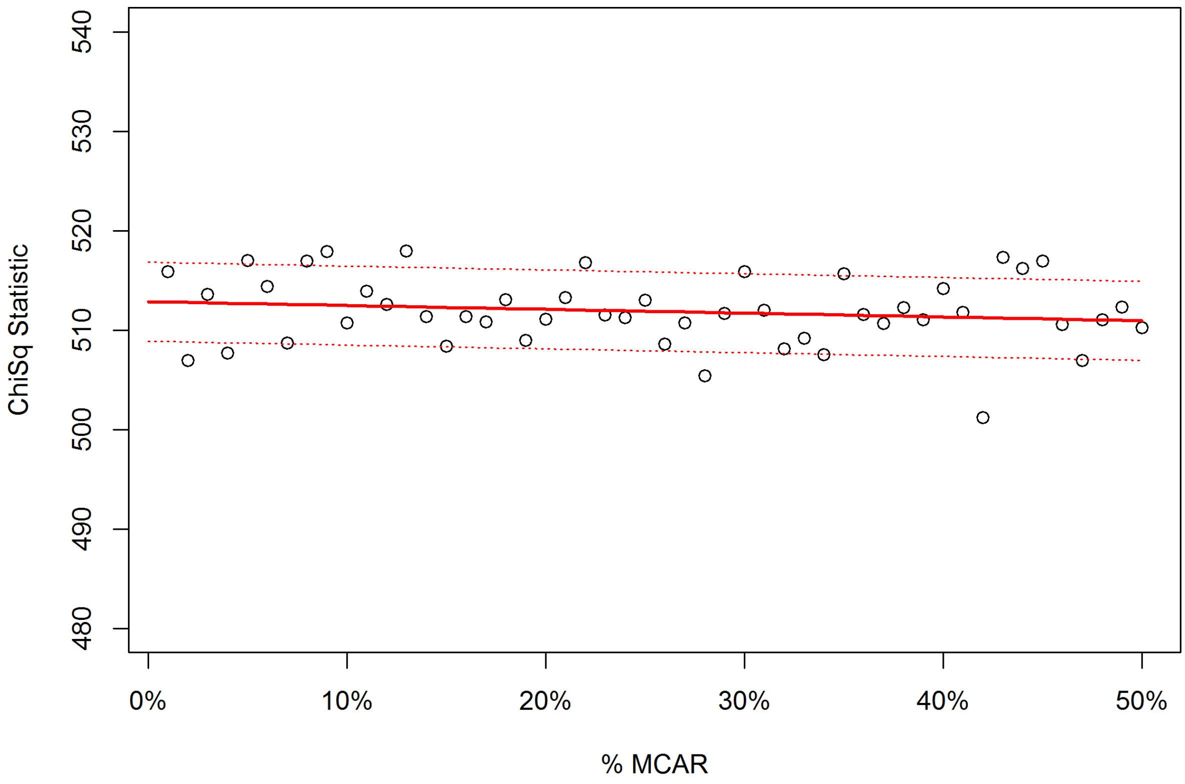

Figure S1 Plot of the relationship between the percentage of MCAR data and the presence of location bias. The points represent marginal means for the observations with the associated percentage of missing data. The regression of %MCAR on the observed Chi-squared statistic is represented by the solid red line; the top and bottom dashed red lines indicate the regression line plus or minus one estimated standard deviation of the estimated effect of %MCAR, respectively. The estimated effect of %MCAR is not statistically significant with a p-value of 0.3341.

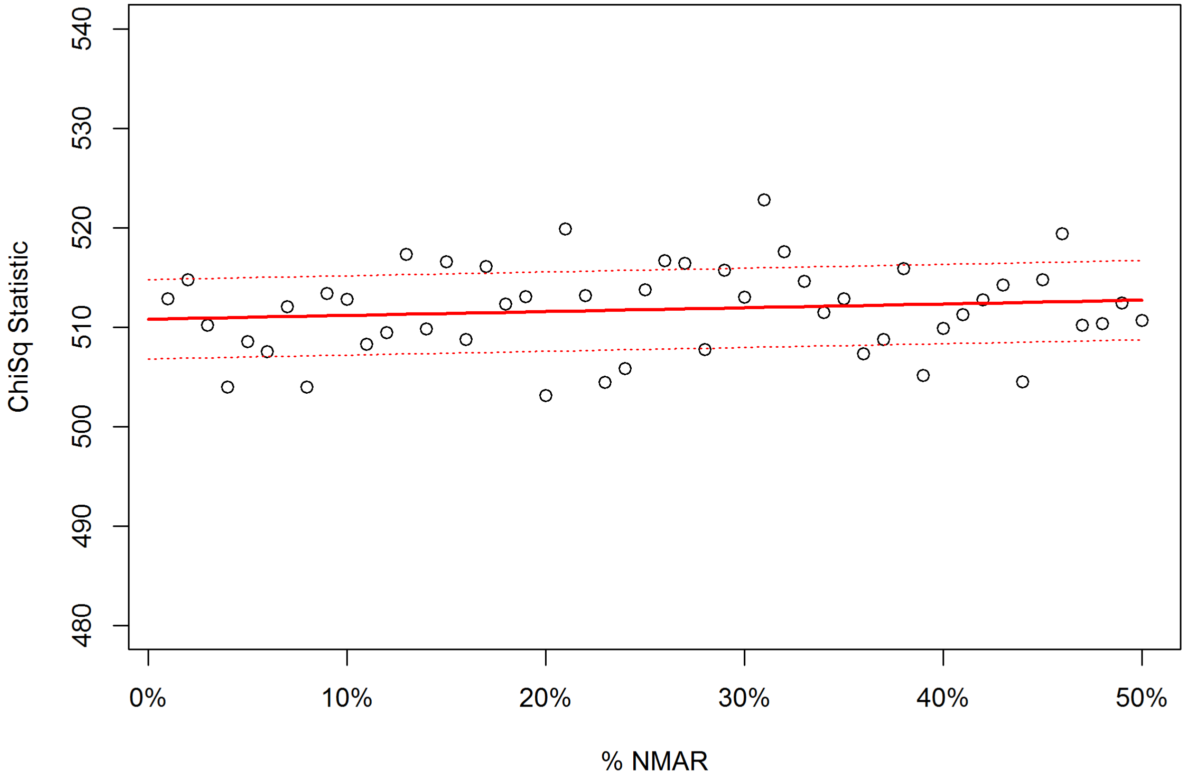

Figure S2 Plot of the relationship between the percentage of NMAR data and the presence of location bias. The points represent marginal means for the observations with the associated percentage of missing data. The regression of %NMAR on the observed Chi-squared statistic is represented by the solid red line; the top and bottom dashed red lines indicate the regression line plus or minus one estimated standard deviation of the estimated effect of %NMAR, respectively. The estimated effect of %NMAR is not statistically significant with a p-value of 0.3369.

Files S2 and S3

Files S2 and S3 are available for download at http://www.g3journal.org/lookup/suppl/doi:10.1534/g3.112.003517/-/DC1.

File S2: R code for Barley Analysis

File S3: Barley Data for Barley Example
